# Tear film lipid layer increase after diquafosol instillation in dry eye patients with meibomian gland dysfunction: a randomized clinical study

**DOI:** 10.1038/s41598-019-45475-7

**Published:** 2019-06-24

**Authors:** Shima Fukuoka, Reiko Arita

**Affiliations:** 1 Omiya Hamada Eye Clinic, 1-169-1 Sakuragicho, Omiya-ku, Saitama, Saitama, 330-0854 Japan; 2 Itoh Clinic, 626-11 Minami-Nakano, Minuma-ku, Saitama, Saitama, 337-0042 Japan; 3 Department of Ophthalmology, University of Tokyo School of Medicine, 7-3-1 Hongo, Bunkyo-ku, Tokyo 113-0033 Japan; 4 Lid and Meibomian Gland Working Group (LIME), 626-11 Minami-Nakano, Minuma-ku, Saitama, Saitama, 337-0042 Japan

**Keywords:** Conjunctival diseases, Corneal diseases, Eyelid diseases

## Abstract

Diquafosol promotes secretion of tear fluid and mucin at the ocular surface and is administered for treatment of dry eye (DE). Tear film lipid layer is secreted from meibomian glands and stabilizes the tear film. We recently showed that diquafosol administration increased lipid layer thickness (LLT) for up to 60 min in normal human eyes. We here evaluated tear film lipid layer in DE patients (*n* = 47) with meibomian gland dysfunction (MGD) before as well as 30, 60, and 90 min after diquafosol administration. One drop of artificial tears or one drop of diquafosol was applied randomly to the eyes of each patient. Diquafosol significantly increased LLT at 30 (*P* < 0.001) and 60 (*P* = 0.042) min and noninvasive tear film breakup time for at least 90 min (*P* < 0.001 at each assessment point). Artificial tears had no such effect. Diquafosol significantly improved the tear interferometric pattern compared with artificial tears (*P* < 0.001 at each assessment point). A single topical administration of diquafosol thus improved LLT and tear film stability in DE patients with MGD, suggesting that diquafosol is a potential treatment not only for aqueous-deficient DE but also for evaporative DE associated with MGD.

## Introduction

Tear film instability is thought to be an important contributor to visual impairment and other symptoms associated with dry eye (DE)^1,2^. The tear film consists of lipids, tear fluid, mucin, and other proteins. The lipid layer of the tear film promotes the rapid spreading of tear fluid after blinking^3,4^, stabilizes the tear film surface^5,6^, and suppresses excessive evaporation of tear fluid^7,8^. Changes to the quality or quantity of tear film components can result in DE, which is classified as either aqueous-deficient (ADDE) or evaporative (EDE)^2^.

Meibomian glands secrete the lipid layer of the tear film, and meibomian gland dysfunction (MGD) thus gives rise to EDE and associated tear film instability^9,10^ and accounts for a substantial proportion of all DE cases^11^. Tear film instability is commonly evaluated on the basis of tear film breakup time^12^, although interference patterns observed by tear interferometry provide important information regarding the nature, thickness, and rupture of the tear film lipid layer. Interferometry thus allows measurement of lipid layer thickness (LLT) and noninvasive tear film breakup time (NIBUT)^2^. Lipid layer deficiency and EDE are largely caused by the obstructive form of MGD (oMGD)^13–18^. LLT has been found to be smaller in patients with ADDE or EDE^3,19^ as well as in those with oMGD^20^ compared with healthy individuals. Patients with severe DE symptoms were also found to have a smaller LLT than those without such symptoms^21^. In addition, NIBUT was shown to be shorter in eyes with MGD than in healthy eyes^10^ as well as to be inversely correlated with the extent of meibomian gland loss^22^. These various findings have indicated that improvement in both LLT and tear film stability is an important goal for the treatment of DE patients with MGD.

Diquafosol is a P2Y_2_ receptor stimulant^23^ and promotes tear fluid^24–28^ and mucin^29,30^ secretion at the ocular surface. A diquafosol sodium ophthalmic solution of 3% (Diquas; Santen Pharmaceutical, Osaka, Japan) is approved and available for DE treatment in Japan, South Korea, Thailand, Vietnam, and China. Animal studies have detected P2Y_2_ receptor expression in meibomian glands as well as in conjunctival and corneal epithelial cells^31,32^. Diquafosol instillation for 2 weeks was found to increase the quantity of intracellular secretory lipid droplets in acinar cells of mouse meibomian glands^33^. A preliminary study also found the quantity of lipid within rabbit meibocytes to be increased by P2Y_2_ agonists *in vitro*^34^. Furthermore, we found that a drop of diquafosol increased LLT for up to 60 min after instillation in healthy human eyes^35^ and that repeated diquafosol instillation for at least 4 months increased meibomian gland area in eyes with oMGD^36^. Diquafosol instillation for 3 months was also shown to reduce the meibum score and meibomian gland loss, with an increase in LLT being apparent at 20 min after instillation, in eyes with DE and MGD^37^. However, the ability of diquafosol to increase ocular lipid secretion and to stabilize the tear film has remained unclear in DE patients with MGD. Using qualitative and quantitative tear interferometry, we have now studied the effects of diquafosol instillation on the lipid layer of the tear film in DE patients with MGD.

## Results

### Demographics as well as subjective symptoms and ocular signs at baseline

A total of 63 patients with DE and MGD was screened for the study. Six patients were excluded because they did not meet the inclusion criteria or met the exclusion criteria before treatment. Fifty-seven subjects were thus enrolled and underwent baseline evaluation. Six individuals met the exclusion criteria on the day of the second visit 1 month later for evaluation of the effects of diquafosol, and four were excluded because of a deviation of experimental time, a missing value, or an LLT_pre_ of >75 nm. Forty-seven subjects (94 eyes) ultimately completed the various tests and their data were analysed (Table 1). The subjective symptom score was 2.9 ± 2.2 for the study patients at baseline. No significant differences were apparent for baseline ocular objective findings between the eyes selected for diquafosol administration and those selected for artificial tear administration (47 eyes in each group) by the Wilcoxon signed-rank test or chi-square test (Table 1).

**Table 1:** Baseline ocular objective parameters for the artificial tear group and the diquafosol group of the dry eye patients with meibomian gland dysfunction (*n* = 47).

Parameter			Artificial tear group	Diquafosol group	*P*
Lid margin abnormalities	Telangiectasia	Upper eyelid (0–3)	1.1 ± 0.7	1.1 ± 0.6	0.66
		Lower eyelid (0–3)	1.0 ± 0.6	1.0 ± 0.7	1.00
	Irregularity	Upper eyelid (0–2)	0.3 ± 0.6	0.3 ± 0.6	0.57
		Lower eyelid (0–2)	0.4 ± 0.6	0.3 ± 0.6	0.083
	Thickness	Upper eyelid (0–2)	0.3 ± 0.5	0.3 ± 0.5	1.00
		Lower eyelid (0–2)	0.3 ± 0.5	0.3 ± 0.5	1.00
	Plugging	Upper eyelid (0–2)	1.5 ± 0.8	1.5 ± 0.8	0.71
		Lower eyelid (0–2)	1.2 ± 0.6	1.2 ± 0.6	0.74
Fluorescein score (0–9)			1.8 ± 1.0	1.8 ± 1.1	0.77
Fluorescein breakup time (s)			2.4 ± 0.9	2.5 ± 1.0	0.49
Schirmer test value (mm)			7.8 ± 6.8	8.2 ± 6.4	0.95
Meiboscore		Upper eyelid (0–3)	1.6 ± 0.8	1.5 ± 0.7	0.24
		Lower eyelid (0–3)	1.5 ± 0.9	1.4 ± 0.9	0.43
		Total (0–6)	3.1 ± 1.4	2.9 ± 1.3	0.13
Meibum grade (0–3)			1.6 ± 0.8	1.5 ± 0.8	0.78
Expressed meibum colour	Upper eyelid [no. (%)]	1 Clear	2 (4.3%)	2 (4.3%)	1.00
		2 Cloudy	34 (72.3%)	34 (72.3%)	
		3 Yellow	11 (23.4%)	11 (23.4%)	
	Lower eyelid [no. (%)]	1 Clear	2 (4.3%)	3 (6.4%)	0.86
		2 Cloudy	35 (74.5%)	33 (70.2%)	
		3 Yellow	10 (21.3%)	11 (23.4%)	
Expressed meibum consistency	Upper eyelid [no. (%)]	1 Oily	11 (23.4%)	7 (14.9%)	0.57
		2 Creamy	31 (66.0%)	35 (74.5%)	
		3 Toothpaste-like	5 (10.6%)	5 (10.6%)	
	Lower eyelid [no. (%)]	1 Oily	10 (21.3%)	6 (12.8%)	0.54
		2 Creamy	32 (68.1%)	36 (76.6%)	
		3 Toothpaste-like	5 (10.6%)	5 (10.6%)	

Data are shown as means ± standard deviations. *P* values were obtained with the Wilcoxon signed-rank test or chi-square test.

### Effect of diquafosol administration on LLT

**Figure 1:**
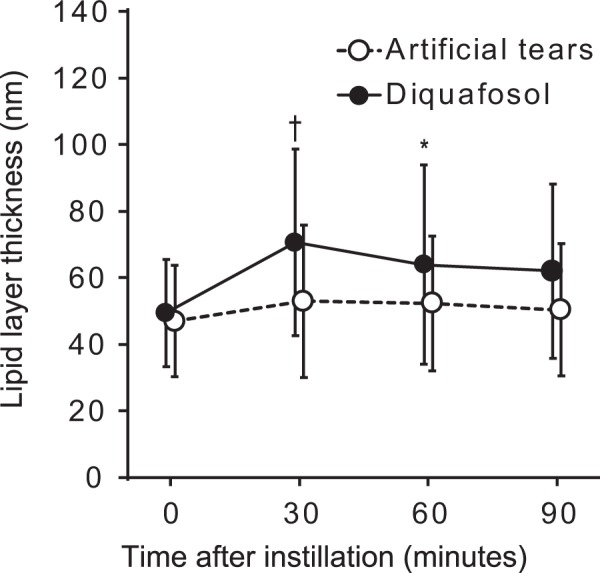
Time course for lipid layer thickness of the tear film before and after artificial tear or diquafosol administration. Data are shown as means ± standard deviations (*n* = 47). *Adjusted *P* < 0.05, ^†^adjusted *P* < 0.001 versus corresponding preadministration (time 0) value (Wilcoxon signed-rank test with Bonferroni’s adjustments for three tests).

One drop of artificial tears or one drop of diquafosol was applied randomly to the eyes of each patient. LLT_pre_ in the control group was not significantly different from that in the diquafosol group (46.9 ± 16.8 versus 49.4 ± 16.2 nm, respectively, *P* = 0.56). LLT was 52.9 ± 22.8, 52.3 ± 20.3, and 50.3 ± 19.8 nm at 30, 60, and 90 min after artificial tear administration, respectively (Fig. 1), with these values not being significantly different from that before administration (adjusted *P* values of 0.26, 0.35, and 1.0, respectively). In marked contrast, LLT increased significantly from 49.4 ± 16.2 nm before to 70.6 ± 28.2 and 63.9 ± 30.0 nm at 30 and 60 min after diquafosol administration, respectively, before declining to 62.0 ± 26.2 nm at 90 min (adjusted *P* values of <0.001, 0.042, and 0.11, respectively). Mixed-effect models for repeated measures (MMRM) also revealed a significant difference in treatment effects on LLT between the two groups (*P < *0.001).

### Effect of diquafosol administration on tear meniscus height (TMH)

**Figure 2:**
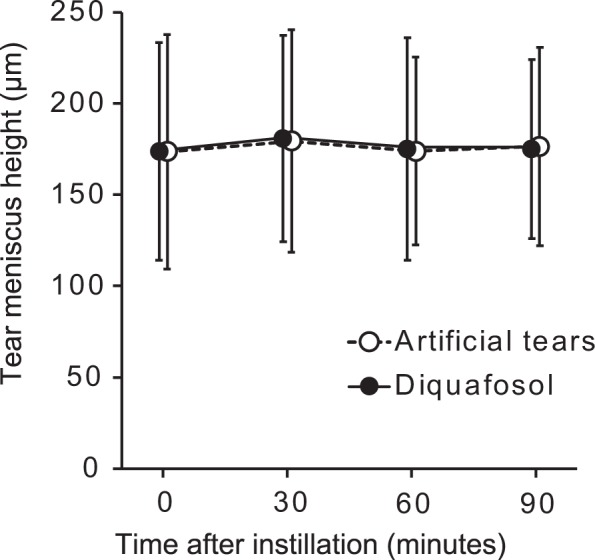
Time course of tear meniscus height before and after artificial tear or diquafosol administration. Data are means ± standard deviations (*n* = 47). No values after administration differed significantly from the corresponding preadministration n (time 0) value (Wilcoxon signed-rank test with Bonferroni’s adjustments for three tests).

TMH_pre_ in the control group was not significantly different from that in the diquafosol group (173.6 ± 64.2 versus 173.6 ± 59.6 μm, respectively, *P* = 0.70). Artificial tears did not significantly affect TMH at 30, 60, or 90 min after instillation compared with TMH_pre_ (179.4 ± 61.0, 173.9 ± 51.6, and 176.5 ± 54.3 μm, with adjusted *P* values of 1.0 at each assessment point) (Fig. 2). TMH was also not significantly affected by diquafosol at 30, 60, or 90 min compared with the initial value (180.6 ± 56.5, 175.2 ± 60.9, and 175.2 ± 49.1 μm, with adjusted *P* values of 0.85, 1.0, and 1.0, respectively). No significant difference in treatment effects on TMH was apparent between the two groups using MMRM (*P* = 0.96).

### Effect of diquafosol administration on NIBUT

**Figure 3:**
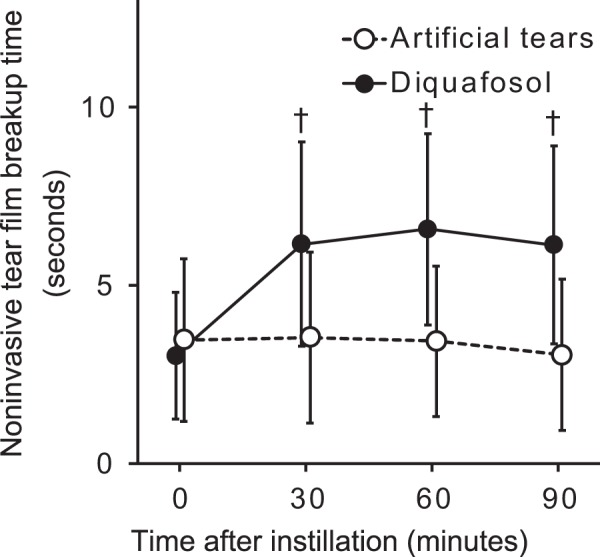
Time course for noninvasive breakup time of the tear film before and after artificial tear or diquafosol administration. Data are shown as means ± standard deviations (*n* = 47). ^†^Adjusted *P* < 0.001 versus corresponding preadministration (time 0) value (Wilcoxon signed-rank test with Bonferroni’s adjustments for three tests).

NIBUT_pre_ in the control group was not significantly different from that in the diquafosol group (3.5 ± 2.3 versus 3.0 ± 1.8 s, respectively, *P* = 0.51). NIBUT was 3.5 ± 2.4, 3.4 ± 2.1, and 3.0 ± 2.1 s at 30, 60, and 90 min after artificial tear administration, respectively (Fig. 3), with these values not differing significantly from the initial value (adjusted *P* values of 1.0, 1.0, and 0.93, respectively). In marked contrast, NIBUT increased significantly from 3.0 ± 1.8 s before to 6.1 ± 2.9, 6.6 ± 2.7, and 6.1 ± 2.8 s at 30, 60, and 90 min after diquafosol administration, respectively (adjusted *P* values of <0.001 at each assessment point). MMRM also revealed a significant difference in treatment effects on NIBUT between the two groups (*P* < 0.001).

### Effect of diquafosol administration on tear interference pattern

**Figure 4:**
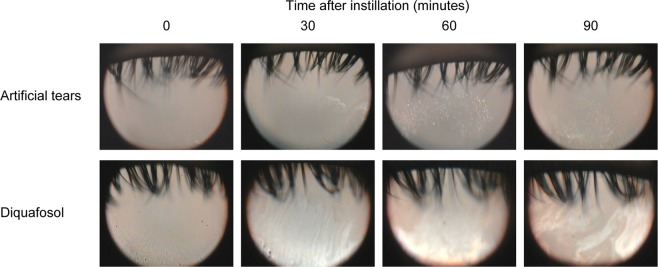
Representative sequential interferometric images of the tear film obtained with the DR-1α tear interferometer before and after artificial tear or diquafosol administration. The subject was a 48-year-old man with dry eye and meibomian gland dysfunction who received a single drop of artificial tears in his right eye and a single drop of diquafosol in his left eye. Before instillation, both eyes manifested a grayish amorphous interferometric image without a fringe (class 2, evaporative dry eye type), corresponding to an unstable tear film with a thin lipid layer but with sufficient tear fluid. Noninvasive breakup time (NIBUT) increased from 2 s before to 5, 7, and 7 s at 30, 60, and 90 min after diquafosol administration, respectively. The interferometric pattern also changed to a monotonous gray fringe (class 0, normal type) after diquafosol administration, corresponding to a stable tear film with a thicker lipid layer. Artificial tear administration did not alter the tear interferometric pattern, but NIBUT showed a transient increase from 2 s before to 3, 4, and 2 s at 30, 60, and 90 min after administration.

**Figure 5:**
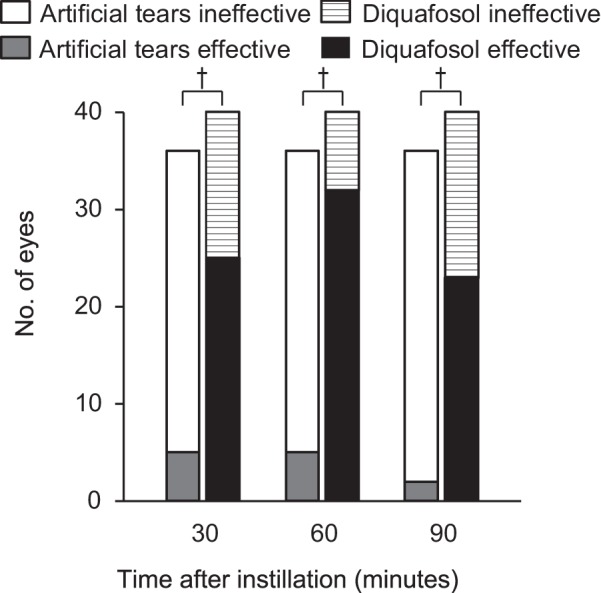
Time course of interferometric pattern improvement after artificial tear or diquafosol administration. Eyes of interferometric class 2 (evaporative dry eye type) before administration were categorized as showing an improvement (that is, treatment was effective) if the interference pattern changed to class 0 (normal type) or class 1 (aqueous-deficient dry eye type). The numbers of eyes of class 2 before administration were 36 and 40 out of 47 in the control group and the diquafosol group, respectively. ^†^*P* < 0.001 versus the control group at each assessment time point (Fisher’s exact test).

Representative tear interferometric images for before and after administration of artificial tears or diquafosol are shown in Fig. 4. Before instillation, 11 eyes (23.4%) were assigned to class 0 (normal type) and 36 eyes (76.6%) to class 2 (EDE type) regarding tear interference pattern in the control group, whereas 6 eyes (12.8%), 1 eye (2.1%), and 40 eyes (85.1%) were assigned to classes 0, 1 (ADDE type), and 2, respectively, in the diquafosol group. No significant difference was observed in the class distribution between the two groups (*P* = 0.26). The proportion of eyes in class 2 that showed an improvement in interference pattern after diquafosol instillation (25 [62.5%], 32 [80.0%], and 23 [57.5%] of 40 eyes) was significantly greater than that after instillation of artificial tears (5 [13.9%], 5 [13.9%], and 2 [5.6%] of 36 eyes) at 30, 60, and 90 min, respectively (*P* < 0.001 at each assessment point of time) (Fig. 5).

### Effect of diquafosol administration on subjective ocular symptoms

No significant difference in visual analogue scale (VAS) scores before instillation was apparent between the control group and the diquafosol group for the following subjective symptoms: ocular fatigue (32.8 ± 29.9 versus 30.0 ± 28.9, *P* = 0.64), discharge (7.8 ± 15.4 versus 7.3 ± 13.8, *P* = 0.76), foreign body sensation (20.5 ± 30.6 versus 17.9 ± 27.7, *P* = 0.76), dryness (33.5 ± 32.7 versus 31.1 ± 32.6, *P* = 0.79), uncomfortable sensation (21.6 ± 31.0 versus 19.4 ± 28.4, *P* = 0.89), pain (12.0 ± 24.2 versus 7.5 ± 17.6, *P* = 0.92), epiphora (11.8 ± 21.3 versus 10.4 ± 17.4, *P* = 0.85), itching (13.4 ± 18.2 versus 13.5 ± 18.6, *P* = 0.84), redness (6.3 ± 11.4 versus 7.3 ± 14.1, *P* = 0.75), heavy sensation (13.4 ± 23.0 versus 12.9 ± 23.3, *P* = 0.79), and glare (12.7 ± 24.9 versus 13.1 ± 23.6, *P* = 0.92). MMRM revealed a significant difference in treatment effects on VAS scores for ocular fatigue, dryness, itching, redness, and heavy sensation between the two groups (*P* values of 0.005, 0.014, 0.001, 0.007, and <0.001, respectively, with these symptoms improving to a greater extent after diquafosol administration), but no significant difference in treatment effects on VAS scores for discharge, foreign body sensation, uncomfortable sensation, pain, epiphora, and glare (*P* values of 0.19, 0.44, 0.075, 0.20, 0.15, and 0.050, respectively).

### Adverse events

No serious adverse events were apparent at any time during the study in either the diquafosol or control group.

## Discussion

Our study investigated the efficacy of single-drop administration of diquafosol and artificial tears on the lipid layer of the tear film in DE patients with MGD with the use of qualitative and quantitative interferometry. Our results demonstrate that diquafosol significantly increased LLT for 60 min after instillation and significantly improved tear film stability for at least 90 min, whereas artificial tears had no such effects. Diquafosol also significantly ameliorated subjective symptoms including ocular fatigue, dryness, itching, redness, and heavy sensation. These findings suggest that topical diquafosol administration may promote lipid secretion from meibomian glands of patients with DE and MGD, and that it is therefore a promising therapeutic option for such patients.

Our finding that diquafosol instillation increased LLT for 60 min suggests that diquafosol promotes lipid secretion as well as fluid^24–28^ and mucin^29,30^ secretion at the ocular surface. We previously found that a single topical diquafosol administration elicited a significant increase in LLT for up to 60 min in healthy human eyes^35^. Previous studies also found that one-drop administration of diquafosol increased tear meniscus area and height as measured by anterior-segment spectral-domain optical coherence tomography at 5 and 10 min after instillation compared with baseline in DE patients^28^ as well as increased the central lower tear meniscus radius of curvature for up to 30 min after administration in healthy human eyes^27^ and at 15 min after administration in Sjögren’s syndrome patients^38^. In our present study, TMH was not increased at 30, 60, or 90 min after diquafosol instillation, although it is possible that it increased transiently after instillation but returned to the initial value before the 30-min time point. We measured noninvasive tear film parameters at 30, 60, and 90 min after drop instillation given that we were interested in the duration of the LLT increase induced by diquafosol. We therefore chose to measure TMH noninvasively by interferometry rather than to perform an invasive procedure such as Schirmer’s test before and after drop administration. We were not able to obtain data at 15 min after instillation because of the time required to complete all the measurements. A significant temporary increase in the concentration of mucin-like substances (sialic acid) in tears was detected at 5 min but not at 15 min after diquafosol administration in healthy human eyes^39^. The mucin 5AC concentration in tears was found to peak at 15 min after diquafosol administration in a rat keratoconjunctivitis sicca model^40^. Data are not available for the time course of mucin secretion after one-drop diquafosol instillation in DE or MGD patients. The persistence of the LLT increase elicited by diquafosol was thus longer than that in tear volume or tear sialic acid concentration. The effect of diquafosol on the lipid layer may therefore be independent of its effects on tear fluid volume or mucin secretion. The duration of P2Y_2_ receptor stimulation by diquafosol in meibomian gland cells might be longer than that in conjunctival goblet cells, and the distribution and turnover of lipid may differ from those of mucin at the ocular surface.

Our study showed that diquafosol significantly improved NIBUT and the tear interferometric pattern for at least 90 min after instillation. These parameters of tear film stability were determined with the DR-1α instrument, which allows noninvasive assessment of a wide area of the ocular surface and analysis of the dynamic nature and temporal instability of the tear film as reflected by NIBUT, the time between the last blink and the appearance of the first lipid layer discontinuity^10,12,41^. After diquafosol instillation, 57.5% to 80.0% of eyes in class 2 showed an improvement in interferometric pattern to class 0 or class 1. The balance of tear film components is thought to be critical for maintenance of the tear film, with loss of such balance giving rise to DE^10^. NIBUT and tear interferometric pattern are affected by several factors, including the quantity and quality of tear film lipid, fluid, and mucin as well as the ocular surface condition and blinking.

Diquafosol is a P2Y_2_ receptor stimulant^23^ and induces both aqueous^24–28^ and mucin^29,30^ secretion at the ocular surface. P2Y_2_ receptors have been detected in the epithelium not only of the conjunctiva and cornea but also of meibomian gland acini and ducts by *in situ* hybridization analysis of rabbit and monkey eyes^31^. Immunostaining also revealed P2Y_2_ receptor expression in the ductal epithelium but not in sebaceous cells of rat meibomian glands^32^. Diquafosol instillation for 2 weeks was found to increase the amount of intracellular secretory lipid droplets in acinar cells of mouse meibomian glands^33^. A preliminary *in vitro* study has also suggested that the number of secretory lipid droplets in rabbit meibomian gland cells increases after diquafosol treatment^34^. In contrast, uridine 5’-triphosphate (UTP), a natural P2Y_2_ receptor agonist, was found to have no effect on human meibomian gland epithelial cells *in vitro*^42^. This discrepancy may have resulted from differences in species or culture conditions. UTP is chemically and metabolically unstable^43^, however, and diquafosol, or P^1^, P^4^-di(uridine 5′-)tetraphosphate tetrasodium salt, was synthesized as a derivative of UTP to improve stability and potency. The stability of diquafosol was found to be ~10 times that of UTP at the mucosal surface of human nasal epithelial cells^43^. We previously found that meibomian gland area was increased in oMGD patients after repeated diquafosol instillation^36^. Instillation of 3% diquafosol for 3 months was also found to improve the meibum score and meibomian gland area, with an increase in LLT being apparent at 20 min after administration, in eyes with DE and MGD^37^. Collectively, these various findings suggest that the diquafosol-induced increase in LLT in our present study was due to P2Y_2_ receptor stimulation in meibomian gland cells. It is therefore likely that diquafosol induces meibum secretion from meibocytes, although further research is necessary to confirm this notion.

Among topical treatments for MGD, lipid emulsion eyedrops^44–46^, lubricant ointment^47^, and a liposomal spray^48^ were found to increase the lipid layer of the tear film. We previously found that diquafosol administration four times daily for not less than 4 months significantly improved meibomian gland area in oMGD patients^36^, and that a single diquafosol application elicited a significant increase in LLT for up to 60 min in healthy eyes^35^. Our present findings now indicate that topical diquafosol administration stimulates meibum secretion from meibomian glands not only in healthy eyes, but also in eyes with DE and MGD. Diquafosol is thus a candidate treatment not only for ADDE but also for EDE associated with MGD.

There are several limitations of the present study. First, the mechanism underlying the increase in tear film LLT induced by diquafosol was not investigated. Second, we measured LLT, TMH, and tear film stability every 30 min after instillation, given that assessment of these parameters is easily influenced by other ocular examinations and a sufficient time interval is required between each set of measurements. We were not able to confirm an increase in TMH after diquafosol instillation, as mentioned above. Third, the effects of long-term repeated diquafosol administration were not examined. A previous study detected a significant LLT increase at 20 min after a single diquafosol administration only after the administration of diquafosol six times a day for 3 months, not at baseline or after treatment for 1 or 2 months, in DE and MGD patients^37^. We also found that diquafosol administration increased LLT in DE and MGD patients, but we determined the time course of this effect and found that it was evident without the need for prior repeated administration. We also performed noninvasive measurements by interferometry to show that a single diquafosol administration improved the balance of the lipid and fluid components of the tear film, without an effect on TMH.

Our results show that one topical administration of diquafosol significantly increased tear film LLT, improved tear film stability, and ameliorated subjective ocular symptoms in DE patients with MGD. Our findings suggest that diquafosol promotes lipid secretion from meibomian glands not only of healthy human subjects but also of patients with DE and MGD. Larger controlled clinical trials will be needed to confirm these effects of diquafosol in such patients.

## Methods

### Study design and subjects

We designed a single-centre, prospective, randomized, masked trial to compare diquafosol or artificial tear administration in paired eyes of DE patients with bilateral MGD. Adult patients with DE and MGD were recruited from September 2016 to March 2017 at Itoh Clinic. Inclusion criteria for both eyes were as follows: (1) the presence of DE symptoms; (2) an initial LLT of ≤75 nm measured with the LipiView tear interferometer (Johnson & Johnson Vision, Jacksonville, FL, USA)^49^; (3) the presence of ocular surface defects as revealed by fluorescein staining; (4) a tear film breakup time determined with fluorescein (FBUT) of ≤5 s or a Schirmer test value of ≤5 mm^50^; (5) at least one lid margin abnormality from among telangiectasia, irregularity, and anterior or posterior replacement of the mucocutaneous junction; and (6) plugged meibomian gland orifices and poor meibum expression^51^. Exclusion criteria included Stevens-Johnson syndrome, ocular pemphigoid, infection, active ocular allergy, daily contact lens wear, punctal plugging, excessive meibomian lipid secretion, no meibum expression from eyelids as evaluated with Arita meibomian gland expressor forceps (Katena, Denville, NJ, USA), eye surgery within the prior 3 months, systemic or ocular diseases that possibly affect tear film, and a history of diquafosol allergy.

The subjects included 47 patients (33 men and 14 women, total of 94 eyes) aged 20 to 76 years (mean ± standard deviations, 48.1 ± 13.0 years). This research adhered to the tenets of the Declaration of Helsinki, and the study was approved by the Institutional Review Board of Itoh Clinic and was registered in the UMIN-CTR database (UMIN000024041, registered on 13 September 2016). The subjects provided written informed consent before study entry.

### First visit: baseline examination

After a 2-week washout period to prevent the influence of any other topical medication, the baseline analyses were performed sequentially, within 15 min in most instances, as follows: (1) Participants were asked about the presence of 14 subjective ocular symptoms: ocular fatigue, discharge, foreign body sensation, dryness, uncomfortable sensation, sticky sensation, pain, epiphora, itching, redness, heavy sensation, glare, excessive blinking, and history of chalazion or hordeolum. Symptoms were scored from 0 to 14 according to the number present, as previously described^52^. (2) Lid margin abnormalities in the upper and lower eyelids were detected and graded by slitlamp microscopy, as previously described^53^. Telangiectasia was graded from 0 (none) to 3 (severe). Irregularity, thickness, and plugging were graded from 0 (none) to 2 (severe). (3) Corneal and conjunctival fluorescein staining was graded from 0 to 9^54^. (4) FBUT was measured three times consecutively after 1 μL of 1% fluorescein dye administration, and the mean of the three values was obtained. (5) Schirmer’s test was performed for 5 min without topical anaesthesia with the use of a Schirmer strip (Whatman no. 41; Showa, Tokyo, Japan). (6) A meiboscore of 0 (no loss) to 3 (loss of more than two-thirds of the gland area) was determined for each eyelid with a noninvasive meibography system (SL-D701 DC-4 BG-5; Topcon, Tokyo, Japan)^55^, with a total meiboscore of 0 to 6 for each eye being obtained by summing the upper and lower eyelid scores. (7) Meibum grade of 0 (clear meibum easily expressed) to 3 (no meibum expression, even with firm pressure) was assessed semiquantitatively according to the difficulty degree of meibum expression by the application of digital pressure to the upper tarsus^16^. (8) Both upper and lower eyelids were compressed with Arita meibomian gland expressor forceps under topical anaesthesia, and the colour of expressed meibum was assessed as 1 (clear), 2 (cloudy), or 3 (yellow). The expressed meibum consistency was also assessed as 1 (oily), 2 (creamy), or 3 (toothpaste-like).

### Second visit: LLT, TMH, NIBUT, tear interference imaging, and subjective ocular symptoms after diquafosol or artificial tear administration

To circumvent the possible influence of other tests on tear film parameter evaluation by quantitative and qualitative interferometry, we studied the effects of diquafosol and artificial tear administration on LLT, TMH, NIBUT, and tear interference pattern at 4 weeks after the baseline examination. Each participant received a single drop of 3% diquafosol (Diquas, Santen) in one eye and a single drop of preservative-free artificial tears (Soft Santear, Santen) in the other eye as a control. The two medications were administered to the right or left eye for each participant in a random manner using permuted-block randomization with a block size of 4 and sequentially numbered opaque sealed envelopes, and the participants and researchers were masked to treatment assignment throughout the trial. An allocation manager (intellim, Tokyo, Japan) generated the random allocation sequence, R.A. enrolled participants, and S.F. applied eyedrops to participants. To ensure equivalent time intervals for the evaluations after drop instillation, we separately timed the two test drop instillations using two stopwatches. LLT, TMH, NIBUT, tear interference, and subjective symptoms were sequentially measured both just before as well as 30, 60, and 90 min after administration as follows: (1) LLT of each eye was quantified with the LipiView instrument^49^. (2) TMH^56^, NIBUT, and tear interference pattern^10^ for each eye were determined with the DR-1α tear interferometer (Kowa, Aichi, Japan)^57,58^. Each eye was assigned to one of three classes on the basis of the combination of interferometric pattern and NIBUT: class 0 (normal type: monotonous gray or multicoloured interferometric fringe with a stable tear film [NIBUT of ≥5 s]), class 1 (ADDE type: multicoloured interferometric fringe and an unstable tear film [NIBUT of <5 s], corresponding to insufficient tear fluid but a thick lipid layer), and class 2 (EDE type: grayish amorphous interferometric image without a fringe and an unstable tear film [NIBUT of <5 s], corresponding to a thin lipid layer but sufficient tear fluid), as previously described^10^. (3) Participants were asked to record subjective ocular symptoms (ocular fatigue, discharge, foreign body sensation, dryness, uncomfortable sensation, pain, epiphora, itching, redness, heavy sensation, and glare) for each eye separately according to VAS scores of 0 (no symptom) to 100 (maximum conceivable symptom), as previously described^59^.

Subjects were instructed to avoid strong blink or rubbing their face before or during examination, and they were monitored for compliance. The temperature and relative humidity during the measurements were 22.5° ± 1.8 °C and 44.2 ± 8.6%, respectively. LLT, TMH, NIBUT, and VAS values at each assessment point of time after administration were compared with those measured before (pre) administration. Eyes of interferometric class 2 before drop instillation that were assigned to class 0 or class 1 after instillation were considered to have undergone an improvement in condition.

### Statistical analysis

The basis of the sample size calculation was as follows: for LLT, a 32.4-nm difference in the mean change after administration between the diquafosol group and the control group, with a corresponding standard deviation value of 31.7 nm; for NIBUT, a 2.6-s difference in the mean change between the two groups after treatment, with a corresponding standard deviation value of 2.4 s. These changes were estimated by results of a pilot study with 30 eyes of 15 subjects in each group. Given these assumptions, we estimated that the sample size requirement would be 43 eyes in each group for a power of >80% and a significant difference at α = 0.017 to ensure an overall type I error rate of 0.05 according to a modified Bonferroni procedure. With a predicted dropout rate of 30%, the required sample size was thus 62 patients.

The data were found to be nonnormally distributed with the Shapiro-Wilk test (*P* < 0.05), and nonparametric testing was selected. The Wilcoxon signed-rank test was used to compare baseline values between the diquafosol group and the control group as well as LLT, TMH, or NIBUT between before and after administration. Given the repeated measurements, MMRM was used to control for preadministration assessment as well as treatment and time effects. The changes in LLT, TMH, NIBUT, and VAS scores were analyzed by MMRM for treatment. The chi-square test was used to compare the colour or consistency of expressed meibum at baseline as well as interferometric class distribution before instillation between the diquafosol group and the control group. The improvement in interferometric pattern was compared between the control group and the diquafosol group at each assessment point of time after administration with Fisher’s exact test. The primary end point was LLT before and after administration, and the secondary end point of the study was NIBUT before and after administration. Bonferroni’s adjustments were used to correct for multiple testing. Statistical analysis was performed with SAS 9.4 software (SAS, Cary, NC, USA). Data are shown as means ± standard deviations. All statistical tests were two-sided, and a P value of <0.05 was considered statistically significant.

## Data Availability

The data sets generated and analysed during the current study are available from the corresponding author on reasonable request.
